# Dissipation of six fungicides in greenhouse-grown tomatoes with processing and health risk

**DOI:** 10.1007/s11356-016-6260-x

**Published:** 2016-03-09

**Authors:** Magdalena Jankowska, Piotr Kaczynski, Izabela Hrynko, Bozena Lozowicka

**Affiliations:** Laboratory of Pesticide Residues, Plant Protection Institute—National Research Institute, Chelmonskiego 22, 15-195 Bialystok, Poland

**Keywords:** Pesticides in tomatoes, Processed tomato pesticides, Fungicides in tomatoes, Dissipation of fungicides, Fungicide dissipation in tomatoes

## Abstract

Greenhouse studies were conducted to evaluate the dissipation rate kinetics and estimate the behavior of selected pesticides after washing, peeling, simmering, and canning of tomato expressed as processing factor (PF). Two varieties (*Marissa* and *Harzfeuer*) were treated by six fungicides: azoxystrobin, boscalid, chlorothalonil, cyprodinil, fludioxonil, and pyraclostrobin at single and double dose and risk assessment defined as hazard quotient was performed. The QuEChERS method was used for sample preparation followed by liquid chromatography coupled with tandem mass spectrometry (LC-MS/MS). The dissipation of fungicides approximately fitted to a first-order kinetic model, with half-life values ranging from 2.49 and 2.67 days (cyprodinil) to 5.00 and 5.32 days (chlorothalonil) for Marissa and Harzfeuer variety, respectively. Results from processing studies showed that treatments have significant effects on the removal of the studied fungicides for both varieties. The PFs were generally less than 1 (between 0.01 and 0.90) and did not depend on variety. The dietary exposure assessed based on initial deposits of application at single and double dose on tomatoes and concentration after each process with PF correction showed no concern to consumer health. Our results would be a useful tool for monitoring of fungicides in tomatoes and provide more understanding of residue behavior and risk posed by these fungicides.

**Electronic supplementary material:**

The online version of this article (doi:10.1007/s11356-016-6260-x) contains supplementary material, which is available to authorized users.

## Introduction

Tomatoes (*Lycopersicon esculentum* Mill.) belong to widely grown fruiting vegetables, and they are currently available for various purposes. The list of tomato cultivars includes about 25,000 varieties (Hixson 2015), and they are diversified in terms of skin color, size, shape, leaf type, and disease resistance code. This crop is susceptible to a number of diseases, thus fungicides have been widely used to control fungal pathogens in greenhouse systems. Some tomato varieties (*Better Boy*, *Celebrity*, *Granadero*, *Red Chief*, *Marissa*, *Erophily*, *Matias*, *Swanson*, *Isabel*, *Kiveli*, *Sesenta*, *Genaros*, *Jury*) are disease resistant, signifying that the plant is immune to a certain disease such as *Alternaria stem canker*, *Fusarium wilt*, *Fusarium* races 1, 2, and 3; *Nematodes*, *Tobacco mosaic virus*, *Stemphylium* gray leaf spot, *Verticillium wilt*.

Fungicides from different groups have been widely used pre- and post-harvest to control fungal tomato pathogens (Matyjaszczyk 2015). Among them, members of the anilinopyrimidine, benzimidazole, carboxamide, chloronitrile, strobilurin, and tiazole family provide good control of tomato diseases. They have different modes of action such as systemic (absorbed through the leaves, stems, or roots) or contact (stay on the surfaces of plants), and they move in various ways after they come in contact with the plant.

To control pesticide residues in vegetables, sensitive and reliable confirmatory methods are necessary (Han et al. 2013) to determine trace amounts these compounds. GC-MS/MS and LC-MS/MS have become valuable techniques in multi-residue analysis. They are currently the most efficient confirmatory tool for discriminating residues at ultra-trace levels (Ucles et al. 2014; Pico et al. 2007).

Pesticide residue monitoring study carried in tomatoes in European Union show that the most frequently detected groups are fungicides over the last years. Mainly dithiocarbamates, boscalid, pyraclostrobin, cyprodinil, fludioxonil, pyrimethanil, and chlorothalonil (EFSA Journal 2015) are detected which have been reported to be capable of causing endocrine disruption and embryotoxic, carcinogenic, and teratogenic effects (PPDB Pesticide Properties Database). The EU set tolerances (maximum residue limits, MRLs) for chlorothalonil, boscalid, cyprodinil, and pyraclostrobin in tomatoes: 6, 3, 1, and 0.3 mg/kg, respectively (EU Pesticide MRLs Database 2013). In contrast, the MRLs are very restrictive for baby food, and they are set at the level of 0.01 mg/kg. However, there are no MRLs for the related processed commodities, such as tomato paste or juice. This gap may be attributed to the lack of processing study data of tomatoes. Therefore, it is necessary to obtain the residues of these pesticides during washing, peeling, juicing, simmering, and sterilization. The fate of a given pesticide needs to be also evaluated to determine dissipation kinetics of fungicides in order to adequately characterize the behavior of a pesticide and health risk assessment.

One way to remove pesticide residues from vegetables is their processing. Many researchers have studied the occurrence of pesticide residues in raw tomatoes (Łozowicka et al. 2015; Salghi et al. 2012), but few studies have focused on their behavior caused by canning. For the most part, pesticide residues in vegetables are reduced or concentrated after several processing such as washing, peeling, blanching, cooking, and sterilization (Holland et al. 1994; Kaushik et al. 2009; Timme and Walz-Tylla 2004). Many studies have been performed to determine how much residue can be eliminated by these types of processes (Berrada et al. 2010; Boulaid et al. 2005; Burchat et al. 1998; Rasmusssen et al. 2003; Lee and Jung 2009; Lentza-Rizos and Balokas 2001; Sakaliene et al. 2009). The effect of processing practices on residues is related with both commodity type and pesticide type (Burchat et al. 1998). However, concentration level after processing can sometimes result in a higher residue in food, for example, as a result of water loss (Timme and Walz-Tylla 2004).

Tomatoes are widely consumed all over the world (Certel et al. 2011) because they are one of the richest sources of lycopene, the potent age-defying antioxidant. After passing through various culinary and processing treatments, they are referred as a “functional food” that people should eat more often. Processed tomato fruits such as tomato juice, paste, soup, sauce, and ketchup are an important part of diet for many consumers because they contain the highest concentrations of bioavailable lycopene than fresh tomatoes. Tomato products are also widely used in children’s feeding between the ages of one and three. The highest consumption of tomatoes indicates Italian population, especially toddlers (GEMS/FOOD 2012).

Because of the negative effects of pesticides on human health for consumers, their intakes in tomatoes and its products are necessary to know. Thus, it is essential to evaluate the level of exposure from pesticide residue in food at the point of consumption after different processing (Bonnechere et al. 2012). Additionally, processing factor (PF: the ratio between residues’ concentration in the processed commodity and that in the raw commodity) is the main parameter used in the dietary intake assessment of pesticides in processed agriculture commodities (Ling et al. 2011). Risk assessments (Łozowicka 2015; Łozowicka et al. 2009) and residue experiments for fungicides in tomatoes are required.

The aims of this study were to (1) evaluate dissipation kinetics of selected fungicides in field-treated two varieties of tomatoes conducted in an experimental greenhouse, to (2) investigate changes of selected pesticide residues after several processing methods in both varieties and provide information regarding the processing factor, and to (3) assess the health risk of consumers eating tomatoes with fungicide residues.

## Material and methods

### Analytical standards and solvents

The analytical standards of azoxystrobin, boscalid, chlorothalonil, cyprodinil, fludioxonil, and pyraclostrobin (<99.0 % purity) were obtained from Dr. Ehrenstorfer (Augsburg, Germany). Stock solutions of six pesticides (around 1000 μg/mL) were prepared separately by dissolving an accurately weighed amount of each reference standard in acetone. The combined working standard solutions were generated by serial dilution of the stock solutions with acetonitrile. The working standard solutions were used for the preparation of matrix-matched standards within the concentration range of 0.005–1.0 μg/mL and for the spiking of samples in the validation studies.

All reagents used pesticide residue grade and were obtained from J.T. Baker (Deventer, Holland).

### Choice of plants and fungicides

Tomatoes were selected to this survey because they are highly consumed by adults as well as children, both in fresh and various processed forms. Among many varieties, two different varieties were chosen to compare dissipation behavior and pesticide concentration changes during processing. Variety *Marissa* is very resistant to many pathogens in cultivation in polish conditions while variety *Harzfeuer* is popular as a market tomato.

The investigations were carried out for six different active substances which were selected according to MRL exceeding, frequency, and level of detection in previous years (EFSA Journal 2015). Based on data of our laboratory, during government monitoring control of pesticide residues, positive detections were noted in 36 % of tomato samples (56 samples) from the north-eastern region of Poland in 2010–2014. The most frequently detected pesticides were dithiocarbamates (19 samples) followed by chlorothalonil (16), fludioxonil (14), azoxystrobin (13), cyprodinil (11), and boscalid (9). Multi-residue samples (30) occurred most frequently in combination of azoxystrobin/chlorothalonil, cyprodinil/fludioxonil, chlorothalonil/dithiocarbamates, and boscalid/pyraclostrobin. Thus, according to those results, a list of potential harmful pesticides was established, and it contained the most often occurring fungicides: azoxystrobin, boscalid, chlorothalonil, cyprodinil, fludioxonil, and pyraclostrobin.

### Greenhouse trial

The purpose of the greenhouse experiment was to produce two different tomato varieties (variety *Marissa* and *Harzfeuer*), exposed to the six selected fungicides.

#### Variety characteristics

Variety *Marissa*—variety description: early hybrid with indeterminate growth, cultivation in protected crops or open fields. The plant is vigorous, highly productive, produces uniform fruits of medium size, resistant to storage and transportation. Fruit weight: 150–170 g, fruit color: dark red, number of seeds: 1000 seeds per one tomato. Variety *Harzfeuer*—variety description: German open pollinated variety. Round, slightly oblate beefsteak-type fruit, more acidic then sweet flavor and juicy. Regular leaf. Fruit weight: 70–90 g, fruit color: red-orange, number of seeds: 250 seeds per one tomato.

#### Cultivation of two varieties of tomatoes

Tomato plants of both varieties were cultivated from May to September 2014 in the greenhouse (6 m × 4 m) located in the Plant Protection Institute—National Research Institute in (Bialystok, Podlasie, Poland 53.139° N, 23.159° E) with no previous pesticide applications following recommended agronomic practices. The tomato plants were grown with a plant spacing 0.5 m × 0.5 m. There were three replications for each treatment (single *for dissipation kinetics* and double dose *for processing treatments*). The greenhouse plants were cultivated under controlled conditions with drip irrigation system.

#### Application of the fungicides

The experimental greenhouse plot was divided into six sub-plots with chemical application and one sub-plot for control without pesticide spraying. Treatments were carried out with fungicides: Amistar Opti 480 SC (containing active ingredients (a.i.): 80 g a.i./L azoxystrobin, 400 g a.i./L chlorothalonil; Syngenta), Signum 33 WG (267 g a.i./kg boscalid, 67 g a.i./kg pyraclostrobin; BASF), and Switch 62.5 WG (375 g a.i./kg cyprodinil, 250 g a.i./kg fludioxonil; Syngenta) at fruiting stage (BBCH code: 81–89, ripening of fruit and seed) (Fig. S1). Pesticides were sprayed individually on plants at single (*for dissipation kinetics*) and at double dose as recommended (*for processing treatments*) (Polish Ministry of Agriculture web site) by a specialized operator using knapsack sprayer to ensure sufficient pesticide primary deposit for the following processing. The plants were separated by foil. The temperature in the greenhouse ranged from 14 to 29 °C and humidity ranged from 75 to 100 % from the day of spraying until harvest.

### Sampling procedure

Whole ripened tomato fruits of equal size after removing of stems (about 2 kg of tomatoes) were collected randomly from the control and treated plots of each treatment at 0 (1 h), 1, 2, 3, 5, 8, 11, 14, and 21 days after application of the fungicides at single dose *for dissipation kinetics*. Immediately after collecting, samples were packed in polyethylene bags and brought to the analytical laboratory, chopped and thoroughly mixed. The homogenized samples were stored deep frozen until analysis no longer than 1 month.

**Fig. 1:**
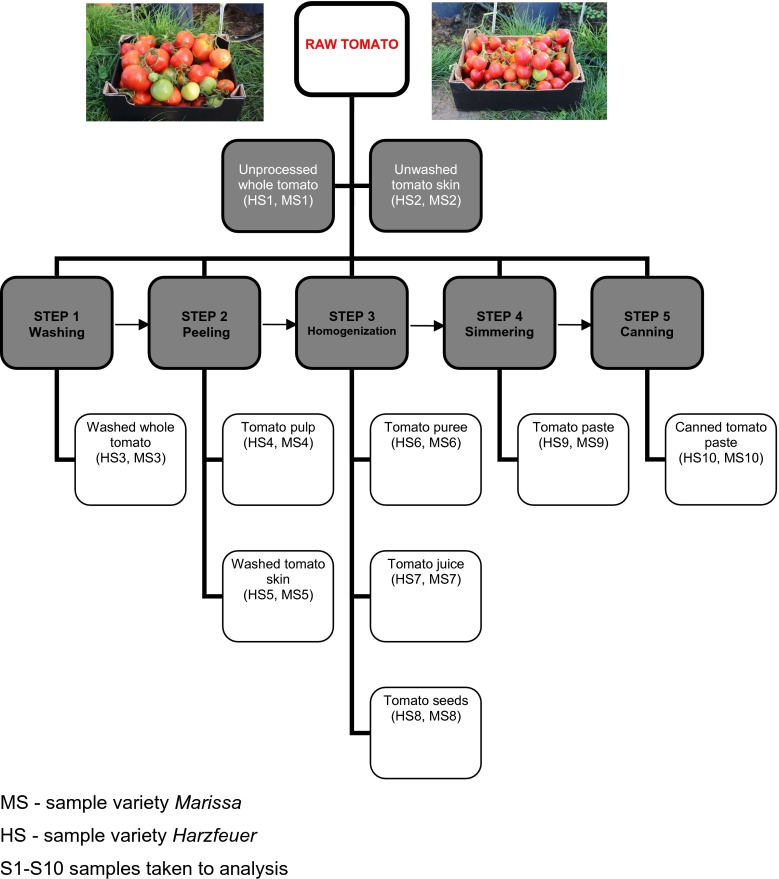
Scheme of tomato processing

To investigate the effects of *processing treatments* on the reduction in residues, about 10 kg each variety of tomatoes were collected 3 days (the pre-harvest interval period of all PPP used according to their labels) after the spraying at double dose. The samples from each treatment were collected separately and were divided into three parts: the first part extracted and analyzed without any processing operation, the second subjected to the peeling process of raw tomatoes, and the third was processed step by step (washing → peeling → homogenization → simmering → canning) to obtain tomato paste (Fig. 1).

### Processing

In general, the production procedures of canned tomato paste included five steps, i.e., washing, peeling, homogenization, simmering, and canning (Fig. 1). In the current study, samples (washed tomato, pulp, skin, puree, juice, seeds, paste, and canned tomato paste: *Marissa* sample (MS) MS2÷MS10 and *Harzfeuer* sample (HS) HS2÷HS10, from different processing steps were collected to determine and investigate the variation of pesticide residues during the processing procedure. As shown in Fig. 1, part of tomatoes was divided from each variety which did not undergo any processing (MS1 and HS1).

The whole fruits of tomatoes were washed under running tap water for 1 min with rubbing with hands and the water was discarded (MS3, HS3). After washing, the whole fruits were peeled off with a knife to obtain tomato pulp (MS4, HS4) and washed tomato skin (MS5, HS5). Also, unwashed tomato skin was taken to analysis (MS2, HS2) before washing. Then, (1) part of the pulp was homogenized to obtain tomato puree (MS6, HS6) and (2) chopped into quarters; the seeds were (MS8, HS8) and excess juice was removed. The juice was homogenized using a blender (MS7, HS7). After that, the tomato pulp was simmered at a temperature of about 80 °C for 20 min (MS9, HS9) and then tomato paste was canned at 120 °C for 20 min (MS10, HS10).

### Extraction and clean up

The samples of tomato were processed and analyzed at the Laboratory of Pesticide Residues, Institute of Plant Protection—National Research Institute, Bialystok, Poland. All samples were extracted by a modified quick, easy, cheap, effective, rugged, and safe (QuEChERS) method according to EN 15662:2008 (European Standard 2008). The QuEChERS method was used for extraction and clean up of fungicide residues in fresh tomato samples and validated for processed tomato products. Representative 10 g of homogenized sample was weighed into a 50 mL PTFE centrifuge tube. Then, 10 mL of acetonitrile were added to the tube and the mixture was placed on a digital Vortex-Mixer (Velp Scientifica, Usmate, Italy) shaker for 5 min at 4500 rpm. Pre-packaged QuEChERS packet of sorbents and salts containing a total of 4 g MgSO_4_, 1 g NaCl, 1 g trisodium citrate dehydrate, and 0.5 g disodium hydrogen citrate sesquihydrate was added, and the tube was immediately shaken for 1 min and then vortexed at full speed for 1 min. Then, the tube was centrifuged using a Rotina 420R centrifuge at 4500 rpm (Hettich) for 10 min at 4500 rpm. The supernatant was transferred to a d-SPE tube containing 150 mg MgSO_4_, 25 mg PSA and then vortexed at full speed for 1 min and centrifuged briefly. Afterward, 1 mL of the upper layer was filtered through 0.2-mm Nylon syringe filters (15 mm diameter, Agela Technologies, China) into the appropriately labeled autosampler vial for LC-MS/MS analysis.

### Instrumentation and LC-MS/MS analytical conditions

**Fig. 2:**
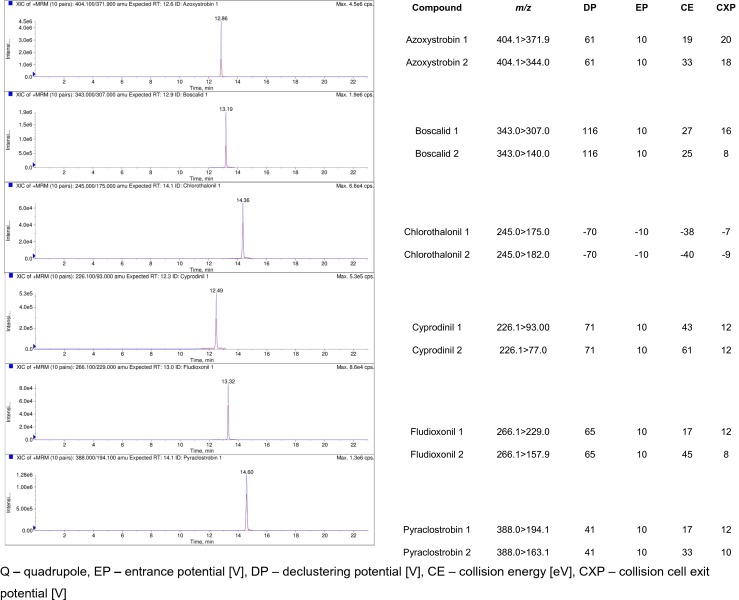
Typical LC-MS/MS chromatograms of fungicides in raw tomato sample (1 and 2: MS/MS transition ions)

An Eksigent Ultra LC-100 (Eksigent Technologies, Dublin, CA, USA) liquid chromatography system was operated at a flow rate of 0.45 mL/min without split chromatographic separation was carried out on a SunFire C_18_ 3.5 μm, 2.1 × 100 mm (Waters) analytical column, maintained at 50 °C during the experiments. The volume injected into the LC-MS/MS system was 10 μL. The binary mobile phase consisted of water with 0.5 % formic acid and 5 mM ammonium formate (phase A) and methanol with 0.5 % formic acid and 5 mM ammonium formate (phase B). The initial composition of 95 % A and 5 % B (*v*/*v*) was held for 2.0 min., followed by linear ramping to 95 % of B in 8 min. and was held for 7 min. After ramping, the mobile phase was returned to the initial composition in 2 min. The total chromatographic run time was 25.0 min. System MS/MS 6500 QTRAP (AB Sciex Instruments, Foster City, CA) was used for mass spectrometric analysis, equipped with an electrospray ionization source (ESI) and atmospheric pressure chemical ionization (APCI). The capillary voltage was maintained at 5000 V for positive ion mode and in case of chlorothalonil at −4500 V for negative ion mode, and the temperature of the turbo heaters was set at 450 °C. As the nebulizer gas (GS1), auxiliary gas (GS2), and curtain gas (CUR), the nitrogen was used at a pressure of 65, 45, and 35 psi, respectively. The nebulizer and collision gas was nitrogen. Optimization of the compounds was performed by injecting individual standard solutions directly into the source (flow injection analysis methods—FIA). Typical LC-MS/MS chromatogram of target fungicides presents Fig. 2.

### Method validation

To analyze the selected pesticides, modified QuEChERS analytical method were used followed by liquid chromatography coupled with a mass spectrometer (LC-MS/MS).

Mean recovery test was performed using spiked blank tomato samples (raw, juice, and paste) at three different concentration levels of selected fungicides (0.005, 0.2, and 1.0 mg/kg). The spiked samples were allowed to settle for 2 h at room temperature prior to the extraction step; this procedure was performed to distribute the pesticide evenly and ensure complete interaction with the sample matrix. The spiked samples were then processed according to the described procedure. The recoveries obtained from the extracted spiked samples were compared with those of the matrix-matched calibration solutions. Calibration curves of the matrix, which were prepared by using aforementioned method, automatically corrected the data for analytical recovery.

The mean recoveries of various concentrations of fungicides in raw tomato, tomato juice, and tomato paste were within 85.53–98.49, 87.53–92.01, and 86.17–96.12 %, respectively (Supplementary data, Table S3). These values were within the range expected for residue analysis. The reproducibility of recovery results, as indicated by relative standard deviations (RSDs) <20 %, confirmed that the method is sufficiently reliable for pesticide analysis in this study (Document No. SANCO/12571/2013 2014).

The limits of quantification (LOQs) were defined as the minimum concentration of the analyte and quantified with acceptable accuracy and precision according to Document No. SANCO /12571/2013 (Document No. SANCO/12571/2013 2014). The limits of detection (LODs) for fungicides were calculated using signal-to-noise criteria (S/N); LOD = 3 (S/N). In this work, the LOQ was estimated to be 0.005 mg/kg and LOD was 0.002 mg/kg for all pesticides.

In addition of the in-house quality assurance programs, during 2006–2014, the laboratory successfully participated in nine inter-laboratory proficiency testing schemes in vegetable matrices organized and run by the Food Analysis Performance Assessment Scheme (FAPAS; Central Science Laboratory in York) and by the European Commission (in the beginning by the University of Uppsala and then by the University of Almeria) with satisfactory results (Supplementary data, Table S4).

### Dissipation kinetics

The degradation kinetics of fungicides in two varieties of tomatoes were determined by plotting residue concentrations against time, and the maximum squares of correlation coefficients found were used to determine the equation of best-fit curves. For all samples, exponential relations were found to apply, corresponding to first-order rate equation. The persistence of fungicides is generally expressed in terms of half-life (t_1/2_) or DT50, i.e., time for the disappearance of pesticide to 50 % of its initial concentration. The rate equation was calculated from the first-order equation: *C*_t_ = *C*_0_e^-kt^, where *C*_t_ represents the concentration of the pesticide residues (mg/kg) at time (days), *C*_0_ represents initial concentration (mg/kg), and *k* is the first-order rate constant (per day) independent of *C*_t_ and *C*_0_. The half-life (*t*_1/2_) was determined from the *k* value for each experiment *t*_1/2_ = ln2/*k*, while the theoretical dissipation time to reach the level of 0.01 mg/kg was calculated according to equation *t*_0.01_ = ln(0.01/*C*_0_)/(−*k*).

### Health risk estimation

The health risk estimation through the comparison of detected fungicide residues with the established acceptable daily intake (ADI) or acute reference dose (ARfD) (JMPR 2006) was calculated. The long-term and short dietary consumer exposure to pesticide residues was estimated by using an EFSA calculation model developed by EFSA (EFSA calculation model Pesticide Residue Intake Model “PRIMo”, revision 2) for two sub-populations, children (2–4 years) and adults (14–80 years). This model based on national food consumption and unit weights and implements internationally agreed risk assessment methodologies to assess the exposure of consumers, accepting consumption at the level of the 97.5 percentile based on the available epidemiological studies carried out for British (PSD 2006) and Italian population (GEMS/FOOD 2012), because data for Polish consumers are available only for general population.

#### Long-term risk assessment

In this study, long-term risk assessment was performed for initial deposits of fungicides obtained at single dose. The acceptable daily intake (ADI) is the estimated amount of a substance in food, expressed on a body weight basis, that can be ingested daily over a lifetime, without appreciable chronic, long-term risk to any consumer. The international estimated daily intake (IEDI) was calculated according to the following formula, where Fi—food-consumption data and RLi—residue level in the commodity: IEDI = Ʃ(Fi × RLi) / *mean*_*body*_*weight*. The long-term risk assessment was performed by calculating the hazard quotient (HQ) by dividing the international estimated daily intake by the relevant acceptable daily intake: HQ_Chronic_ = IEDI/ADI.

#### Short-term risk assessment

Short-term risk was estimated by comparing single intake of the highest detected residue of fungicide (HR) full portion consumption data for the commodity unit (F) to a set volume ARfD. The international estimated short-term intake (IESTI) was calculated for processed samples according to the following formulas (Renwick 2002): IESTI = (F × HR) / *mean*_*body*_*weight* (without correction for PF) and IESTI* = (F × HR*PF) / *mean*_*body*_*weight* (correcting for PF). In short-term risk assessment, HQ was calculated by the equation: HQ_Acute_ = IESTI/ARfD. The assessment of the acute exposure was based on a worst-case scenario, i.e., consumption data for consumers with extreme food consumption habits were combined with the highest residue concentration.

## Results and discussion

### Decline of fungicide residues

**Fig. 3:**
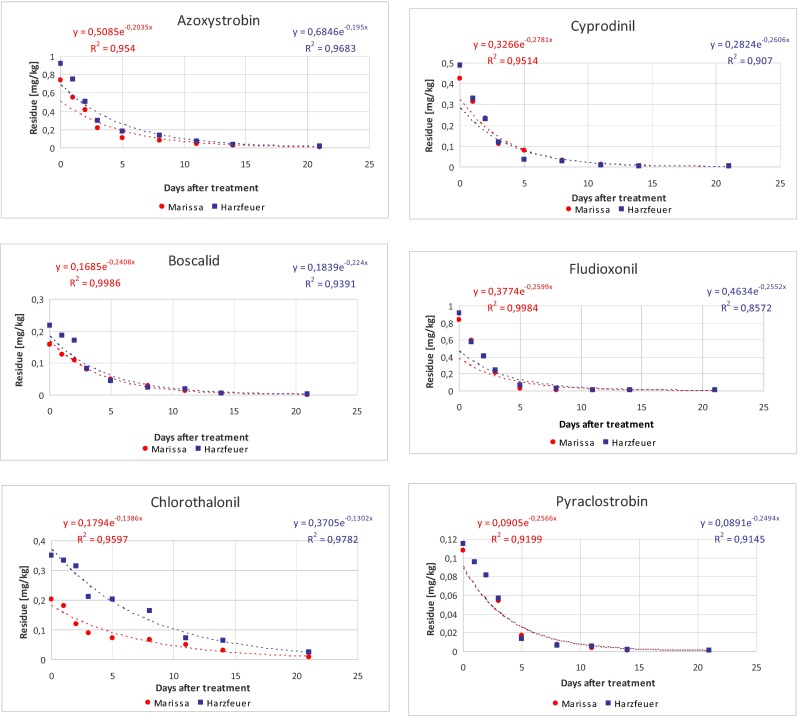
Dissipation kinetics of active substances studied in two varieties of tomatoes

The values of azoxystrobin, boscalid, chlorothalonil, cyprodinil, fludioxonil, and pyraclostrobin residues for two varieties *Marissa* and *Harzfeuer* of tomatoes are shown in Table 1 (a and b), respectively. The average initial residues of six fungicide residues for variety *Marissa* and for variety *Harzfeuer* were in the range 0.158–1.076 and 0.217–1.143 mg/kg, respectively. At the end of the experiment, the concentration of pesticides decreased to 0.090–0.541 and 0.121–0.568 mg/kg, which indicated that up to 99 % of the initial deposits dissipated over the 21 days of the experiment. The dissipation rate of residues was initially faster but slowed down over time (Fig. 3), showing a non-linear trend that fitted with the first-order kinetic model. Figure 3 shows the regression equations and correlation coefficient for fungicides in both tomato varieties.

**Table 1:** Fate of fungicides studied in two varieties of tomatoes: (a) variety *Marissa* and (b) variety *Harzfeuer*

Days after treatment	Azoxystrobin		Boscalid		Chlorothalonil		Cyprodinil		Fludioxonil		Pyraclostrobin	
	Mean C ± SD	D %	Mean C ± SD	D %	Mean C ± SD	D %	Mean C ± SD	D %	Mean C ± SD	D %	Mean C ± SD	D %
	*n* = 3		*n* = 3		*n* = 3		*n* = 3		*n* = 3		*n* = 3	
(a) Variety *Marissa*												
0 (1 h)	0.741 ± 0.076	–	0.158 ± 0.017	–	0.203 ± 0.022	–	0.425 ± 0.044	–	0.835 ± 0.085	–	0.108 ± 0.013	–
1	0.552 ± 0.060	25.51	0.128 ± 0.014	18.99	0.180 ± 0.022	11.33	0.313 ± 0.032	26.35	0.588 ± 0.060	29.58	0.095 ± 0.011	11.25
2	0.415 ± 0.048	43.99	0.108 ± 0.012	31.65	0.120 ± 0.013	40.89	0.236 ± 0.025	44.47	0.407 ± 0.042	51.26	0.081 ± 0.009	24.54
3	0.215 ± 0.023	70.99	0.081 ± 0.009	48.73	0.090 ± 0.011	55.67	0.111 ± 0.013	73.88	0.211 ± 0.026	74.73	0.054 ± 0.006	49.72
5	0.112 ± 0.016	84.89	0.049 ± 0.006	68.99	0.073 ± 0.008	64.04	0.081 ± 0.009	80.94	0.027 ± 0.003	96.77	0.017 ± 0.002	84.48
8	0.083 ± 0.009	88.80	0.028 ± 0.004	82.28	0.066 ± 0.007	67.49	0.032 ± 0.004	92.47	0.014 ± 0.002	98.32	0.007 ± 0.001	93.68
11	0.043 ± 0.005	94.20	0.012 ± 0.002	92.41	0.050 ± 0.006	75.37	0.011 ± 0.002	97.41	0.010 ± 0.002	98.80	<LOQ	<99
14	0.028 ± 0.004	96.22	0.006 ± 0.001	96.20	0.031 ± 0.004	84.73	<LOQ	>99	0.009±	98.92	<LOQ	>99
21	0.010 ± 0.003	98.65	<LOQ	>99	0.008 ± 0.001	96.06	<LOQ	>99	<LOQ	>99	<LOQ	>99
*k*	0.2035		0.2408		0.1386		0.2781		0.2599		0.2566	
*t*_1/2_	3.41		2.88		5.00		2.49		2.67		2.70	
*t*_0.01_	21.16		11.46		21.72		13.48		17.03		9.26	
(b) Variety *Harzfeuer*												
0 (1 h)	0.917 ± 0.094	–	0.217 ± 0.025	–	0.351 ± 0.040	–	0.488 ± 0.051	–	0.909 ± 0.094	–	0.114 ± 0.015	–
1	0.750 ± 0.080	18.21	0.186 ± 0.021	14.29	0.332 ± 0.035	5.41	0.328 ± 0.0034	32.79	0.570 ± 0.059	37.29	0.095 ± 0.010	16.71
2	0.504 ± 0.052	45.04	0.171 ± 0.019	21.20	0.315 ± 0.033	10.26	0.232 ± 0.025	52.46	0.404 ± 0.042	55.56	0.081 ± 0.009	29.22
3	0.293 ± 0.031	68.05	0.084 ± 0.090	61.29	0.211 ± 0.025	39.89	0.121 ± 0.014	75.20	0.243 ± 0.026	73.27	0.057 ± 0.006	50.31
5	0.179 ± 0.020	80.48	0.044 ± 0.005	79.72	0.204 ± 0.022	41.88	0.037 ± 0.004	69.42	0.072 ± 0.008	92.08	0.013 ± 0.001	88.80
8	0.135 ± 0.015	85.28	0.022 ± 0.003	89.86	0.164 ± 0.017	53.28	0.028 ± 0.003	94.26	0.025 ± 0.003	97.25	0.006 ± 0.001	94.66
11	0.072 ± 0.008	92.15	0.017 ± 0.002	92.17	0.071 ± 0.008	79.77	0.009 ± 0.001	98.16	0.012 ± 0.001	98.68	0.005 ± 0.001	95.19
14	0.039 ± 0.005	95.75	0.005 ± 0.001	98.16	0.063 ± 0.007	82.05	0.005 ± 0.001	99.18	0.008 ± 0.001	99.12	<LOQ	<99
21	0.015 ± 0.001	98.36	<LOQ	>99	0.024 ± 0.003	93.16	<LOQ	>99	0.007 ± 0.001	99.23	<LOQ	>99
*k*	0.195		0.224		0.1302		0.2597		0.2552		0.2494	
*t*_1/2_	3.55		3.09		5.32		2.67		2.72		2.78	
*t*_0.01_	23.17		13.74		27.33		14.97		17.67		9.77	

*Mean C* concentration (mg/kg), *SD* standard deviation, *n* number of replicates, *D* dissipation, *k* rate constant (days-1), *t*_1/2_ half-life time (days), *t*_0.01_ theoretical time to reach the level of 0.01 mg/kg

The half-life values (*t*_1/2_), theoretical dissipation time (*t*_0.01_) to reach the concentration of 0.01 mg/kg and dissipation rate constants (*k*) of the six fungicides are summarized in Table 1. The half-life values of the pesticides were 2.49–5.00 days for variety *Marissa* and 2.67–5.32 days for variety *Harzfeuer*. The shortest half-life time for cyprodinil and the longest for chlorothalonil were noted in both varieties. The theoretical dissipation time was 9.26–21.72 and 9.77–27.33 days for variety *Marissa* and *Harzfeuer*, respectively. Pyraclostrobin was the fungicide which the fastest reach the level of 0.01 mg/kg while chlorothalonil the slowest. Residues dissipated below quantification limits at the twenty-first days except chlorothalonil and azoxystrobin.

The results of the present study were consistent with findings found in the literature. The half-life values of other fungicides have been previously reported to be 2.2 days for metalaxyl in cucumbers (Ramezani and Shahriari 2015) and 2.7 days for iprovalicarb in cabbage heads (Maity and Mukherjee 2009). It has been shown (Fig. 3) that most fungicide residues dissipated faster in variety *Marissa* that in variety *Harzfeuer*. This can be explained by the differences in size. Tomato fruits of variety *Harzfeuer* are about two times smaller than variety *Marissa*.

The pre-harvest intervals (PHIs) for all the studied fungicides were establish by the Polish government at 3 days in greenhouse-grown tomatoes. Roughly 49.72–74.73 % of the initial deposits of pesticides were lost after PHIs for variety *Marissa*, while the dissipation was 39.89–75.20 % for variety *Harzfeuer*. Additionally, the longest dissipation time at residue level 0.01 mg/kg was obtained for chlorothalonil 21.72 and 27.33 days for Marissa and Harzfeuer variety, respectively. Chlorothalonil is a fairy persistent fungicide with long residual activity. Based on these observations, longer safety waiting periods are suggested for chlorothalonil in tomatoes, especially in the case of food intended for children.

### Unprocessed tomato samples

The unprocessed tomato samples obtained from greenhouse trial with initial deposits of fungicides were necessary to calculate the processing factors which describe the efficiency of food processing in terms of reducing the pesticide residue level. With obtained concentrations of raw tomato samples (Table 2), processing factors have been calculated to estimate the level of pesticide exposure at the point of consumption after processing.

**Table 2:** Concentrations of fungicides after various processing steps: (a) variety *Marissa* and (b) variety *Harzfeuer*

Sample	Pesticide	Azoxystrobin	Percent reduction	Boscalid	Percent reduction	Chlorothalonil	Percent reduction	Cyprodinil	Percent reduction	Fludioxonil	Percent reduction	Pyraclostrobin	Percent reduction
		Mean C ± SD		Mean C ± SD		Mean C ± SD		Mean C ± SD		Mean C ± SD		Mean C ± SD	
		*n* = 3		*n* = 3		*n* = 3		*n* = 3		*n* = 3		*n* = 3	
(a) Variety *Marissa*													
MS1	Raw tomato	0.184 ± 0.022	–	0.377 ± 0.035	–	0.339 ± 0.038	–	0.372 ± 0.039	–	0.210 ± 0.025	–	0.125 ± 0.005	–
MS3	Washed tomato	0.059 ± 0.065	68	0.146 ± 0.015	61	0.151 ± 0.016	55	0.219 ± 0.024	41	0.108 ± 0.013	49	0.089 ± 0.091	29
MS2	Unwashed tomato skin	0.181 ± 0.019	–	0.345 ± 0.035	–	0.333 ± 0.038	–	0.257 ± 0.029	–	0.234 ± 0.026	–	0.158 ± 0.017	–
MS5	Washed tomato skin	0.039 ± 0.005	78	0.092 ± 0.012	73	0.033 ± 0.005	90	0.172 ± 0.018	33	0.038 ± 0.005	84	0.076 ± 0.008	52
MS4	Tomato pulp	0.022 ± 0.003	88	0.044 ± 0.055	88	0.074 ± 0.081	78	0.069 ± 0.076	81	0.065 ± 0.007	69	0.034 ± 0.004	73
MS6	Tomato puree	0.016 ± 0.001	91	0.041 ± 0.005	89	0.073 ± 0.005	78	0.065 ± 0.005	83	0.046 ± 0.005	78	0.013 ± 0.005	90
MS7	Tomato juice	<LOQ	>99	<LOQ	>99	<LOQ	>99	<LOQ	>99	<LOQ	>99	<LOQ	>99
MS8	Tomato seeds	<LOQ	>99	<LOQ	>99	<LOQ	>99	<LOQ	>99	<LOQ	>99	<LOQ	>99
MS9	Tomato paste	0.017 ± 0.001	91	0.037 ± 0.005	90	0.050 ± 0.005	85	0.020 ± 0.001	95	0.019 ± 0.005	91	0.010 ± 0.001	92
MS10	Canned tomato	0.028 ± 0.005	85	0.091 ± 0.005	76	0.005 ± 0.001	99	0.026 ± 0.005	93	0.039 ± 0.005	81	0.019 ± 0.002	85
(b) Variety *Harzfeuer*													
HS1	Raw tomato	0.217 ± 0.026	–	0.420 ± 0.046	–	0.411 ± 0.045	–	0.424 ± 0.045	–	0.301 ± 0.035	–	0.134 ± 0.014	–
HS3	Washed tomato	0.134 ± 0.015	38	0.274 ± 0.028	35	0.239 ± 0.029	42	0.271 ± 0.028	36	0.156 ± 0.018	48	0.120 ± 0.015	10
HS2	Unwashed tomato skin	0.212 ± 0.027	–	0.374 ± 0.040	–	0.417 ± 0.046	–	0.395 ± 0.042	–	0.306 ± 0.035	–	0.206 ± 0.026	–
HS5	Washed tomato skin	0.093 ± 0.095	56	0.097 ± 0.012	74	0.035 ± 0.005	92	0.223 ± 0.027	44	0.070 ± 0.009	77	0.099 ± 0.012	52
HS4	Tomato pulp	0.039 ± 0.006	82	0.087 ± 0.011	55	0.121 ± 0.014	71	0.030 ± 0.005	93	0.099 ± 0.005	67	0.038 ± 0.005	72
HS6	Tomato puree	0.032 ± 0.005	85	0.133 ± 0.005	68	0.116 ± 0.005	72	0.018 ± 0.003	96	0.061 ± 0.005	80	0.018 ± 0.005	87
HS7	Tomato juice	<LOQ	>99	<LOQ	>99	<LOQ	>99	<LOQ	>99	<LOQ	>99	<LOQ	>99
HS8	Tomato seeds	<LOQ	>99	<LOQ	>99	<LOQ	>99	<LOQ	>99	<LOQ	>99	<LOQ	>99
HS9	Tomato paste	0.035 ± 0.007	84	0.052 ± 0.008	88	0.019 ± 0.003	95	0.035 ± 0.005	92	0.049 ± 0.006	84	0.012 ± 0.001	91
HS10	Canned tomato	0.035 ± 0.005	84	0.098 ± 0.005	77	0.005 ± 0.001	99	0.010 ± 0.005	98	0.037 ± 0.004	88	0.014 ± 0.001	90

*Mean C* mean concentrations in mg/kg, *SD* standard deviation, *n* number of replicates

### Effect of processing and processing factors

The level and nature of pesticide residues in food have always been changed during home processing (Li et al. 2011). Several studies have examined the effect of commercial or home processing on pesticide residue removal in fruits and vegetables (Aguilera et al. 2012; Amvrazi 2011; Bonnechere et al. 2012; Keikotlhaile et al. 2010). The processing techniques used in our studies focused on processing of tomatoes, including washing, peeling, homogenization, simmering, and canning. The experiment focused on concentration changes of azoxystrobin, boscalid, chlorothalonil, cyprodinil, fludioxonil, and pyraclostrobin and determination of processing factors (PFs) on each step during tomato paste production.

A processing study was performed to investigate the effect of particular technological steps on the residues of selected fungicides in two varieties of tomato fruits. Many factors could affect the removal of pesticide residue such as chemical property of pesticide, processing procedure, etc.

Analyzed fungicides (Supplementary data, Table S1) belong to various chemical groups, e.g., anilinopyrimidine, carboxamide, chloronitrile, phenylpyrrole, and strobilurin according to Database of University of Hertfordshire (PPDB Pesticide Database) and have different health effects for humans (Supplementary data, Table S2). The effectiveness of each treatment depended on physico-chemical properties of the studied fungicides such as octanol-water partition coefficient (logP), solubility in water (S_w_), boiling point and molecular mass (M), and the mode of action. The concentration changes of fungicide residue in tomatoes after processing were presented in Table 2.

Washing is the first step in most processing methods. The effectiveness of washing in removing of residues depends on many factors (Kaushik et al. 2009) including the location of residue, the age of residue, the water solubility, the lipophilic character of the pesticide, and the washing technique (Holland et al. 1994). The traditional method of washing vegetables to remove debris and dirt has been assumed to reduce pesticide residues (Satpathy et al. 2012). In the present work, raw tomato samples were washed under running tap water. The results indicated that fungicides were reduced by 29–68 % and 10–48 % after washing for *Marissa* and *Harzfeuer* variety, respectively (MS2, HS2). It was noted that removal of contact pesticides like chlorothalonil and fludioxonil were higher in contrast to systemic cyprodinil or pyraclostrobin.

As shown in Table 2, the maximum fungicide residues were obtained from unwashed tomato skins (MS3, HS3). The amount of residues decreased up to 90 and 92 % after the peeling process (MS5, HS5). This result indicated that pesticides were primarily deposited on the tomato skin. Cutin and wax may have important functions in physically protecting tomato fruit from pesticide deposition (Kimbara et al. 2012). A similar finding was studied by (Mourad Boulaid et al. 2005), who found that pyrifenox and tralomethrin residues cannot be detected in peeled tomato samples.

Peeling the tomatoes efficiently removed almost all fungicide residues, two non-systemic fungicides more efficiently compared to four systemic compounds. This was expected, as chlorothalonil and fludioxonil are non-systemic fungicides, making them immobile in plant tissue and therefore located on the outer surface of the peel. Whereas, azoxystrobin, boscalid, cyprodinil, and pyraclostrobin are systemic, making them mobile in plant tissue and penetrating deeper into the plant tissues. These fungicides might also end up in the tomato fruits via xylem and pholem transport from other parts of the plant, therefore being more present in the tomato pulp in addition to peel.

After peeling, tomatoes were cut into quarters, and the seeds and excess juice were removed. The juice was homogenized using a blender to preserve its taste. The data in Table 2 indicated that residues in tomato seeds and juice were below the LOQ in this study. These results may have been caused by the physico-chemical properties of pesticides, including their solubility in water. Fungicides studied are relatively insoluble in water (Supplementary data, Table S1), the solubility in water ranged from 0.81 mg/l for chlorothalonil to 13.00 mg/l for cyprodinil (at 20 °C). Thus, they are hardly transported into the internal parts of tomato juice (MS7, HS7) and tomato seeds (MS8, HS8) because of their low water solubility.

Many researchers have reported about reduction of pesticide concentration in different vegetables. Randhawa et al. (2007) found that peeling reduced 60–67 % of the endosulfan residues in vegetables, whereas washing reduced 15–30 % of these residues. Timme et al. and Burchat et al. reported results for the peeling and the juicing of carrots. According to them, peeling allows the elimination of residues and the juice was less concentrated in pesticide residues than the pulp (Timme and Walz-Tylla 2004; Burchat et al. 1998).

**Fig. 4:**
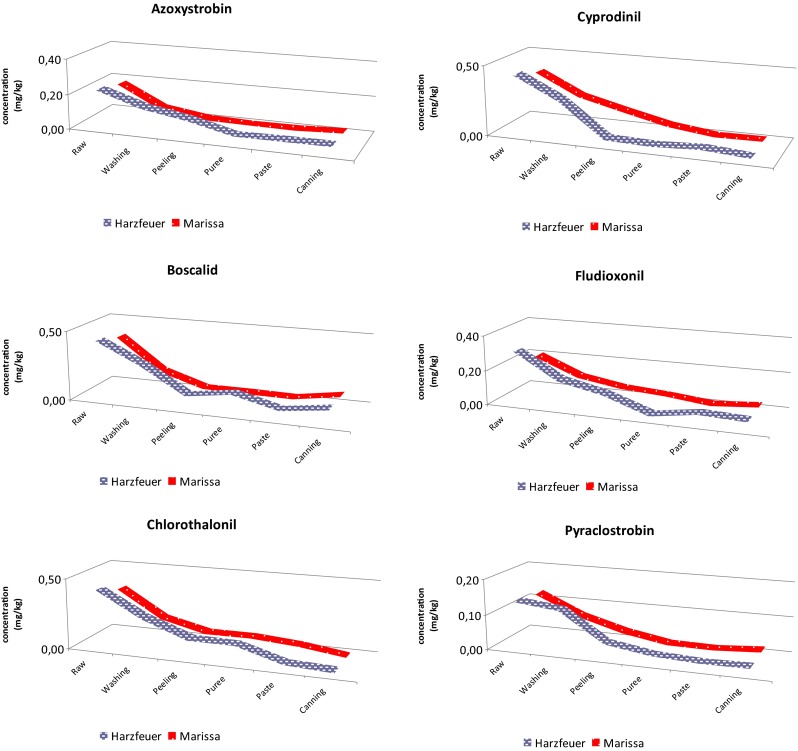
Trends of fungicide content during processing in two varieties of tomatoes

The next process was simmering and was applied to remove excess water from tomato puree. About 50 % of the water in the tomatoes was evaporated. As shown in the Fig. 4, the residues in tomato paste (MS9, HS9) were reduced up to 92 %. The highest removal of initial deposits was observed for pyraclostrobin. Obtained high degradation of pyraclostrobin may be explained by the fact that during thermal processing, the loss of pesticide residues may be through evaporation, co-distillation, and thermal degradation and thus reduce residue levels (Holland et al. 1994).

The last step was canning of tomato paste carried at very high temperature (about 120 °C). As shown in Table 2, the most pesticide residues in tomato paste were lower than those in canned tomatoes (MS10, HS10). This result may be explained by the fact that the pesticides were concentrated as the water evaporated from the tomatoes. However, in our study, one exception was noted. Chlorothalonil indicated almost complete reduction after canning in both varieties, with removal up to 99 %. It can be explained that during heat treatment, some of the pesticides are lost by volatilization or hydrolysis, and some of the compound can also be degraded. Similar findings were obtained by Li et al. (2011), who found that sterilization eliminated the cypermethrin and prochloraz residues.

The processing factor are calculated and considered by the Joint FAO/WHO Meeting on Pesticide Residues (JMPR) as follows (FAO/WHO 2012): PF = residues in processed tomatoes (mg/kg) / residues in raw tomatoes (mg/kg). The PF values below 1 (i.e., reduction factor) indicate a reduction in residues in a processed commodity, whereas the values above 1 (i.e., concentration factor) indicate concentration effects from the processing procedures (Timme and Walz-Tylla 2004). Table 3 shows the calculated PFs for fungicides after processing in both varieties. The PFs were generally less than 1, which indicates residue reduction in the processed tomato commodities. In particular, the general PF for studied fungicides obtained after washing was 0.57, after peeling 0.24, after simmering 0.10, and after canning 0.12. The lowest PF value among the data obtained indicated washing followed by peeling and simmering and thus they played the most important role in effectively removing residues from both varieties of tomatoes.

**Table 3:** Processing factors (PFs) for individual processing steps for six pesticides in two varieties of tomatoes

Fungicide	Variety	PF^a^				
		Washing	Peeling	Homogenization	Simmering	Canning
Azoxystrobin	M	0.32	0.12	0.09	0.09	0.15
	H	0.62	0.18	0.15	0.16	0.16
Boscalid	M	0.39	0.12	0.11	0.10	0.24
	H	0.65	0.45	0.32	0.12	0.23
Chlorothalonil	M	0.45	0.22	0.22	0.15	0.01
	H	0.58	0.29	0.28	0.05	0.01
Cyprodinil	M	0.59	0.19	0.17	0.05	0.07
	H	0.64	0.07	0.04	0.08	0.02
Fludioxonil	M	0.51	0.31	0.22	0.09	0.19
	H	0.52	0.33	0.20	0.16	0.12
Pyraclostrobin	M	0.71	0.27	0.10	0.08	0.15
	H	0.90	0.28	0.13	0.09	0.10
General PF		0.57	0.24	0.17	0.10	0.12

^a^PF for individual processing steps were obtained from samples MS3, HS3 in washing; MS4, HS4 in peeling; MS6, HS6 in homogenization; MS9, HS9 in simmering; MS10, HS10 in canning

Figure 4 shows the trend of fungicide content during processing in both varieties of tomatoes. The general trend of reduction of pesticide residues by certain methods of food processing for a particular active ingredient was noted. Figure 4 shows some differences between varieties, especially in concentrations after washing step. Although, primary deposits of fungicides were higher in variety *Harzfeuer* than in variety *Marissa*, the final concentrations in canned tomato paste were close in both varieties. It could be concluded that proportion peels/pulp was more important and engendered variations between varieties which were leveled during processing.

### Safety evaluation

The value of ADI for azoxystrobin, boscalid, chlorothalonil, cyprodinil, fludioxonil, and pyraclostrobin is 0.2, 0.04, 0.015, 0.03, 0.37, and 0.03 mg/kg, respectively, and ARfD is available only for chlorothalonil and pyraclostrobin 0.60 and 0.03 mg/kg, respectively. Consumption of tomatoes at 97.5 percentile per person is 6.4643 g/kg body weight (bw) for British children and 4.0428 g/kg bw for British adults, whereas for Italian population is 9.1576 g/kg bw for Italian toddlers and 3.0231 g/kg bw for Italian adults.

#### Chronic risk assessment

In the present study, with the first-day concentration of fungicides at recommended dose, the estimated daily intakes were found to be IEDI = 0.69*10^−3^–5.88*10^−3^ (fludioxonil) g/kg body weight/day for children and IEDI = 0.38*10^−3^–3.22*10^−3^ g/kg body weight/day for adults. The calculated percent IEDI and ADI ratios ranged between HQ_Chronic_ = 2.3–15.1 % and HQ_Chronic_ = 0.8–8.3 % for British children and adults, respectively. In contrast for Italian population, toddlers eat about three times more than adults, thus IEDI ranged from 0.98*10^−3^ to 8.32*10^−3^ g/kg body weight/day for toddlers and from 0.32*10^−3^ to 2.74*10^−3^ g/kg body weight/day for adults with hazard quotient values HQ_Chronic_ = 2.1–21.4 % and HQ_Chronic_ = 0.7–7.1 %, respectively. For both population, the lowest HQ_Chronic_ value was for fludioxonil while the highest for chlorothalonil. Chlorothalonil is a compound from chloronitriles and is also considered to be a carcinogen for humans (Supplementary data, Table S2), so it is important to respect pre-harvest intervals of this fungicide to prevent excessive residues on the harvested crop.

#### Acute risk assessment

The dietary exposure was also calculated for initial deposits at double dose. In case of unavailability of ARfD, we accepted the ADI value for calculations. For British population, IESTI ranged from 0.81*10^−3^ to 2.74*10^−3^ g/kg body weight/day for children and IESTI from 0.44*10^−3^ to 1.50*10^−3^ g/kg body weight/day for adults with HQ_Acute_ = 0.4–9.1 % and HQ_Acute_ = 0.2–5.0 %, respectively. While calculated IESTI for Italian children ranged from 0.11*10^−3^ to 3.88*10^−3^ g/kg body weight/day and for Italian adults from 0.38*10^−3^ to 1.28*10^−3^ g/kg body weight/day with HQ_Acute_ = 0.5–12.9 % and HQ_Acute_ = 0.2–4.3 %, respectively. Interestingly for both populations, the lowest HQ_Acute_ value was obtained for chlorothalonil while the highest for cyprodinil. This can be explained by the fact that chlorothalonil has its ARfD value forty times higher than ADI.

#### Intake corrections

The acute intakes (IESTI for British population) obtained for fungicide levels at pre-harvest intervals after double-dose application have been used to intake corrections. The acute risk assessment calculation has been performed for both varieties of tomatoes after each processing treatment. Intakes for British children and adults have been corrected with PFs and are shown in Table 4. After multiplying the assessed intakes with processing factors obtained for pesticides at each step during canned tomato paste production (washing, peeling, homogenization, simmering, and canning). IESTI* for children and adults are presented in Table 4. No significant effects of fungicide residues in tomatoes on human health were observed because the values were relatively low.

**Table 4:** Acute risk assessment for children and adults

Fungicide	IESTI children	IESTI adults	Variety	Washing		Peeling		Homogenization		Simmering		Canning	
				IESTI*	IESTI*	IESTI*	IESTI*	IESTI*	IESTI*	IESTI*	IESTI*	IESTI*	IESTI*
				Children	Adults	Children	Adults	Children	Adults	Children	Adults	Children	Adults
Azoxystrobin	1.19*10^−3^	0.65*10^−3^	M	3.81*10^−4^	2.08*10^−4^	1.43*10^−4^	7.81*10^−5^	1.07*10^−4^	5.86*10^−5^	1.07*10^−4^	5.86*10^−5^	1.78*10^−4^	9.76*10^−5^
	1.40*10^−3^	0.77*10^−3^	H	8.70*10^−4^	4.76*10^−4^	2.52*10^−4^	1.38*10^−4^	2.10*10^−4^	1.15*10^−4^	2.24*10^−4^	1.23*10^−4^	2.24*10^−4^	1.23*10^−4^
Boscalid	2.44*10^−3^	1.33*10^−3^	M	9.50*10^−4^	5.20*10^−4^	2.92*10^−4^	1.60*10^−4^	2.68*10^−4^	1.47*10^−4^	2.44*10^−4^	1.33*10^−4^	5.85*10^−4^	3.20*10^−4^
	2.71*10^−3^	1.48*10^−3^	H	1.76*10^−3^	9.66*10^−4^	1.22*10^−3^	6.69*10^−4^	8.69*10^−4^	4.75*10^−4^	3.26*10^−4^	1.78*10^−4^	6.24*10^−4^	3.42*10^−4^
Chlorothalonil	2.19*10^−3^	1.20*10^−3^	M	6.57*10^−4^	3.60*10^−4^	4.82*10^−4^	2.64*10^−4^	4.82*10^−4^	2.64*10^−4^	3.29*10^−4^	1.80*10^−4^	2.19*10^−5^	1.20*10^−5^
	2.66*10^−3^	1.45*10^−3^	H	1.57*10^−3^	8.58*10^−4^	7.70*10^−4^	4.22*10^−4^	7.44*10^−4^	4.07*10^−4^	1.33*10^−4^	7.27*10^−5^	2.66*10^−5^	1.45*10^−5^
Cyprodinil	2.40*10^−3^	1.31*10^−3^	M	1.42*10^−3^	7.76*10^−4^	4.57*10^−4^	2.50*10^−4^	4.09*10^−4^	2.24*10^−4^	1.20*10^−4^	6.58E-05	1.68*10^−4^	9.21*10^−5^
	2.74*10^−3^	1.50*10^−3^	H	1.75*10^−3^	9.60*10^−4^	1.92*10^−4^	1.05*10^−4^	1.10*10^−4^	6.00*10^−5^	2.19*10^−4^	1.20*10^−4^	5.48*10^−5^	3.00*10^−5^
Fludioxonil	1.36*10^−3^	0.74*10^−3^	M	6.92*10^−4^	3.79*10^−4^	4.21*10^−4^	2.30*10^−4^	2.99*10^−4^	1.63*10^−4^	1.22*10^−4^	6.69*10^−5^	2.58*10^−4^	1.41*10^−4^
	1.94*10^−3^	1.06*10^−3^	H	1.01*10^−3^	5.54*10^−4^	6.42*10^−4^	3.51*10^−4^	3.89*10^−4^	2.13*10^−4^	3.11*10^−4^	1.70*10^−4^	2.33*10^−4^	1.28*10^−4^
Pyraclostrobin	0.81*10^−3^	0.44*10^−3^	M	5.74*10^−4^	3.14*10^−4^	2.18*10^−4^	1.19*10^−4^	8.08*10^−5^	4.42*10^−5^	6.46*10^−5^	3.54*10^−5^	1.21*10^−4^	6.63*10^−5^
	0.87*10^−3^	0.47*10^−3^	H	7.80*10^−4^	4.27*10^−4^	2.43*10^−4^	1.33*10^−4^	1.13*10^−4^	6.16*10^−5^	7.80*10^−5^	4.27*10^−5^	8.66*10^−5^	4.74*10^−5^

IESTI and IESTI* in g/kg body weight/day

Including PFs in the intakes for children was below 1.76*10^−3^, 1.22*10^−3^, 8.69*10^−4^, 3.29*10^−4^, and 6.24*10^−4^ g/kg body weight/day after washing, peeling, homogenization, simmering, and canning, respectively, with HQ_Acute_ below 4.4 %. For adults, IESTI* was reduced to 9.66*10^−4^, 6.69*10^−4^, 4.75*10^−4^, 1.80*10^−4^, and 3.42*10^−4^ g/kg body weight/day after washing, peeling, homogenization, simmering, and canning, respectively, with HQ_Acute_ below 2.4 %. The HQ estimated from acute dietary exposure was above 20 % and after intake correction was reduced to 4 %. This finding indicated that the processing steps obviously reduced pesticide residues and corresponding risks to consumer health.

## Conclusion

In the current work, distribution of azoxystrobin, boscalid, chlorothalonil, cyprodinil, fludioxonil, and pyraclostrobin in two varieties *Marissa* and *Harzfeuer* cultivated in greenhouse were evaluated. The persistence of the fungicides was in the following order: cyprodinil > fludioxonil > pyraclostrobin > boscalid > azoxystrobin > chlorothalonil. The effects of washing, peeling, homogenization, simmering, and sterilization on these fungicide residues in two varieties of tomato were also determined. The concentrations of pesticide residues significantly decreased in canned tomato paste by home processing. The processing factors obtained for a particular combination of fungicide/processing treatment allowed to better understand the removal effects of different pesticide residues in processed tomatoes by washing, peeling, homogenization, simmering, and canning. The reduction of the pesticides depended on the physico-chemical properties and systemic character of the pesticides and allowed to make assumptions to explain the difference in the processing factors for the studied pesticides. The evaluated dietary exposure after correction for PFs of all fungicides indicated no relevant risk to consumers as well children and adults. Therefore, tomato paste did not cause adverse effects on human health, especially for the most vulnerable population small children.

These results provided valuable information regarding the behavior of fungicides during tomato paste production as well as the effective role of technology in removing residues from tomato and reducing health risk of consumers. Reducing the frequency and levels of pesticides in food will build consumer confidence in the safety of fresh produce and is a solid step in the right direction in promoting healthier dietary consumption patterns.

## Electronic supplementary material

Below is the link to the electronic supplementary material.

ESM 1 Table S1. Physico-chemical properties of pesticides used in this experiment. Table S2. Health effects of active substances. Table S3. Validation parameters for tomatoes. Table S4. Analytical quality check in vegetable matrices. Figure S1. Greenhouse scheme. Graphical abstract (DOC 408 kb)
