# Supplementary material for: Dissipation of six fungicides in greenhouse-grown tomatoes with processing and health risk
Source: Environ Sci Pollut Res Int. 2016 Mar 9;23:11885–900. doi: 10.1007/s11356-016-6260-x (PMC4893063; doi:10.1007/s11356-016-6260-x)

**Supplementary data**

**Table S1** Physico-chemical properties of pesticides used in this experiment.

| **Active substance**  Group  Chemical formula | **Structure of active substance and its properties** | **Active substance**  Group  Chemical formula | **Structure of active substance and its properties** |
| --- | --- | --- | --- |
| **azoxystrobin** (PubChem CID: 3034285)  Strobilurin C_22_H_17_N_3_O_5_ | 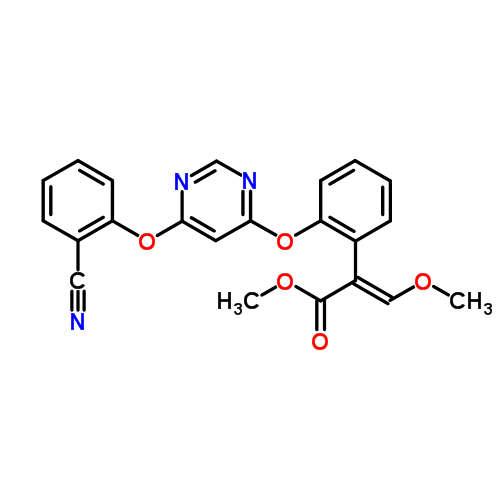  IUPAC name: methyl (E)-2-{2-[6-(2-cyanophenoxy)pyrimidin-4-yloxy]phenyl}-3-methoxyacrylate  Systemic; logP=2.5; Sw=6.7 mg/l; M=403.4 g/mol, pKa not applicable (no dissociation) BCF - low | **cyprodinil**  (PubChem CID: 86367)  Anilinopyrimidine C_14_H_15_N_3_ | 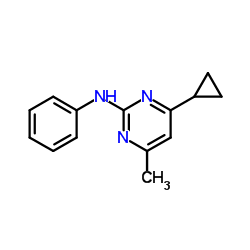  IUPAC name: 4-cyclopropyl-6-methyl-N-phenylpyrimidin-2-amine  Systemic; logP= 4.0; Sw=13.0 mg/l; M=225.29 g/mol, pKa=4.44 (weak base), BCF=393 l/kg |
| **boscalid**  (PubChem CID: 213013)  Carboxamide  C_18_H_12_Cl_2_N_2_O | 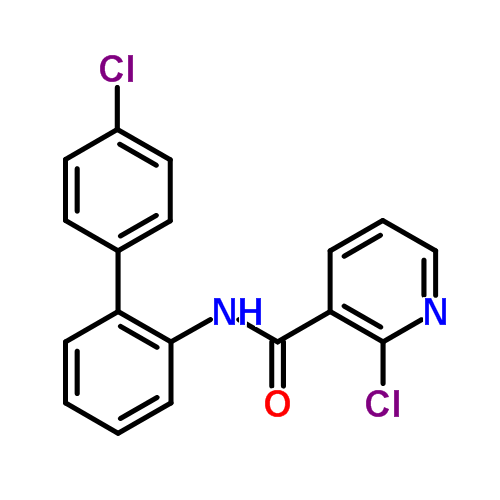  IUPAC name: 2-chloro-N-(4'-chlorobiphenyl-2-yl)nicotinamide  Systemic, logP=2.96; Sw=4.6 mg/l; M=343.21 g/mol, pKa not applicable (no dissociation), BCF=107 l/kg | **fludioxonil**  (PubChem CID: 86398)  Phenylpyrrole  C_12_H_6_F_2_N_2_O_2_ | 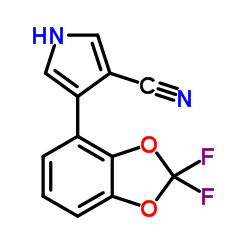  IUPAC name: 4-(2,2-difluoro-1,3-benzodioxol-4-yl)-1H-pyrrole-3-carbonitrile  Non-systemic; logP=4.12; Sw=1.8 mg/l; M=248.19 g/mol, pKa 0, BCF=366 l/kg |
| **chlorothalonil**  (PubChem CID: 15910)  Chloronitrile  C_8_Cl_4_N_2_ | 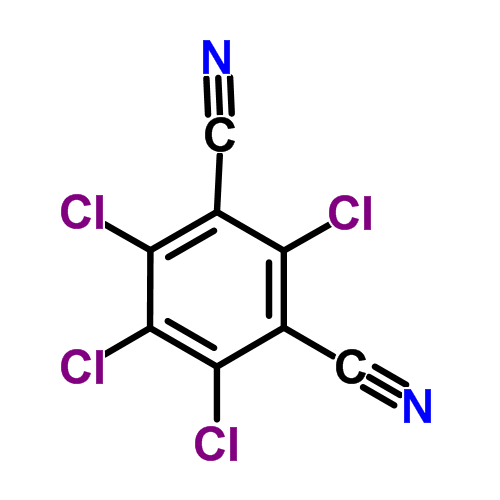  IUPAC name: tetrachloroisophthalonitrile  Non-systemic, logP=2.94; Sw=0.81 mg/l; M=265.91 g/mol, pKa not applicable (no dissociation), BCF=100 l/kg | **pyraclostrobin**  (PubChem CID: 6422843)  Strobilurin  C_22_H_21_ClN_2_O_4_ | 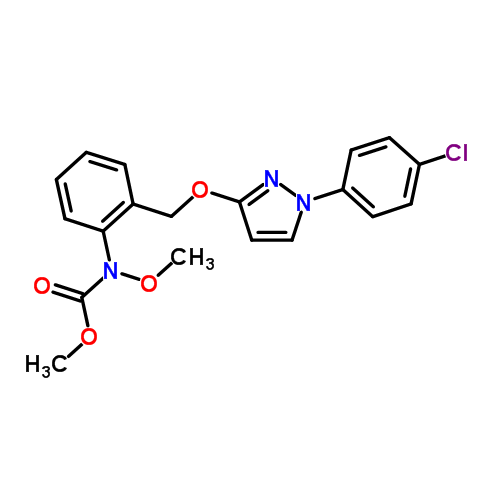  IUPAC name: methyl (2E)-2-(2-{[3-(4-chlorophenyl)-1-methylpyrazol-5-yl]oxymethyl}phenyl)-3-methoxyacrylate  Systemic, logP=3.99; Sw=1.9 mg/l; M=412.87 g/mol, pKa not applicable (no dissociation), BCF=706 l/kg |

logP - octanol-water partition coefficient at pH 7, 20 ^o^C; Sw - solubility in water at 20^o^C (mg/l), M - Molecular mass, pKa - dissociation constant at 25^o^C, BCF- bio-concentration factor [l/kg]

**Table S2.** Health effects of active substances.

|  | **Carcinogen** | **Mutagen** | **Endocrine disrupter** | **Reproduction / development effects** | **Cholinesterase inhibitor** | **Neurotoxicant** | **Respiratory tract irritant** | **Skin irritant** | **Eye irritant** |
| --- | --- | --- | --- | --- | --- | --- | --- | --- | --- |
| Azoxystrobin | 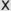 | - | - | 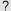 | 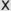 | 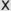 | - | 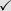 | 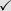 |
| Boscalid | 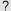 | - | 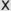 | 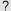 | 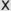 | 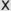 | 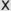 | 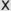 | 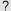 |
| Chlorothalonil | 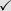 | 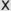 | 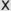 | 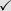 | 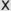 | 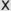 | 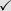 | 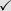 | 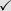 |
| Cyprodinil | 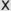 | 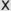 | - | 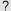 | 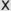 | 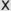 | 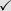 | 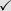 | 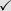 |
| Fludioxonil | 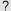 | - | - | 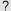 | 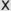 | 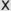 | 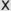 | 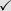 | 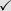 |
| Pyraclostrobin | 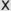 | - | - | 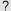 | 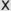 | 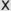 | 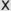 | 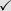 | 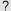 |


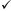
: Yes, known to cause a problem

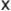
: No, known not to cause a problem

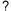
: Possibly, status not identified
- : No data

**Table S3** Validation parameters for tomatoes.

| **Active substance** | **Fortification level (mg/kg)** | **Tomato** | | **Tomato**  **juice** | | **Tomato**  **pulp** | |
| --- | --- | --- | --- | --- | --- | --- | --- |
|  |  | Mean recoveries (%) | RSD (%) | Mean recoveries (%) | RSD (%) | Mean recoveries (%) | RSD (%) |
| azoxystrobin | 0.005 | 97.52 | 13 | 95.21 | 13 | 96.59 | 14 |
|  | 0.2 | 98.49 | 10 | 94.90 | 10 | 97.54 | 18 |
|  | 1.0 | 98.45 | 11 | 90. 15 | 11 | 91.43 | 16 |
| boscalid | 0.005 | 93.28 | 16 | 93.80 | 16 | 97.84 | 10 |
|  | 0.2 | 95.15 | 12 | 92.10 | 12 | 99.25 | 11 |
|  | 1.0 | 96.22 | 13 | 91.22 | 13 | 94.17 | 13 |
| chlorothalonil | 0.005 | 91.38 | 15 | 90.38 | 16 | 97.44 | 15 |
|  | 0.10 | 91.52 | 12 | 90.52 | 18 | 91.29 | 11 |
|  | 1.00 | 97.20 | 18 | 92.20 | 17 | 97.24 | 12 |
| cyprodinil | 0.005 | 86.10 | 12 | 89.10 | 12 | 86.17 | 10 |
|  | 0.2 | 85.53 | 11 | 87.53 | 10 | 86.56 | 11 |
|  | 1.0 | 86.77 | 19 | 89.77 | 11 | 87.78 | 17 |
| fludioxonil | 0.005 | 96.00 | 10 | 90.02 | 12 | 96.01 | 15 |
|  | 0.2 | 94.78 | 17 | 91.87 | 14 | 95. 18 | 16 |
|  | 1.0 | 96.49 | 14 | 95.40 | 15 | 91.40 | 12 |
| pyraclostrobin | 0.005 | 93.06 | 16 | 92.64 | 13 | 90.01 | 17 |
|  | 0.2 | 91.59 | 18 | 90.12 | 11 | 95.19 | 16 |
|  | 1.0 | 94.61 | 15 | 92. 01 | 10 | 96.12 | 12 |

**Table S4** Analytical quality check in vegetable matrices.

|  | **Pesticide/ Proficiency test**  **matrix and code** | **Assigned value (mg/kg)** | **Laboratory results (mg/kg)** | **z-score** |
| --- | --- | --- | --- | --- |
| **Paprika Homogenate** EUPT-FV16, 2014 | | | | |
| 1 | Acetamipryd | 0.632 | 0.450 | -1.2 |
| 2 | Acrinatrine | 0.261 | 0.280 | 0.3 |
| 3 | Buprofezin | 0.467 | 0.450 | -0.1 |
| 4 | chlorothalonil | 2.230 | 3.200 | 1.7 |
| 5 | Chlorpyrifos | 3.680 | 3.700 | 0.0 |
| 6 | cypermethrin | 0.528 | 0.620 | 0.7 |
| 7 | Cyprodinil | 0.591 | 0.560 | -0.2 |
| 8 | Diazinon | 0.084 | 0.090 | 0.3 |
| 9 | difenoconazole | 0.953 | 1.100 | 0.6 |
| 10 | endosulfan alfa | 1.160 | 0.950 | -0.7 |
| 11 | endosulfan beta | 0.975 | 0.900 | -0.3 |
| 12 | Fenhexamid | 0.861 | 0.800 | -0.3 |
| 13 | Fludioxonil | 0.241 | 0.300 | 1.0 |
| 14 | lambda cyhalothrin | 0.077 | 0.080 | 0.2 |
| 15 | Pyrimicarb | 0.731 | 0.700 | -0.2 |
| 16 | Pyrideben | 0.151 | 0.130 | -0.6 |
| 17 | tetraconazole | 0.104 | 0.100 | -0.2 |
| **Potato homogenate** EUPT-FV15, 2013 | | | | |
| 1 | azoxystrobin | 0.203 | 0.204 | 0.0 |
| 2 | chlorothalonil | 0.160 | 0.155 | -0.1 |
| 3 | chlorpropham | 1.700 | 1.292 | -1.0 |
| 4 | cypermethrin | 0.100 | 0.105 | 0.2 |
| 5 | Diazinon | 0.195 | 0.204 | 0.2 |
| 6 | Iprovalicarb | 0.090 | 0.105 | 0.7 |
| 7 | Pencycuron | 0.269 | 0.265 | -0.1 |
| 8 | Prochloraz | 0.058 | 0.061 | 0.2 |
| 9 | procymidone | 0.110 | 0.126 | 0.6 |
| 10 | thiabendazole | 1.710 | 1.462 | -0.6 |
| **Potato homogenate** EUPT-SRM8, 2013 | | | | |
| 1 | Captan | 1.010 | 0.920 | -0.36 |
| 2 | Dicofol | 1.030 | 0.860 | -0.66 |
| 3 | Folpet | 1.320 | 1.350 | 0.09 |
| **Cucumber Purée** FAPAS, 2010 | | | | |
| 1 | Chlorpyrifos | 0.0837 | 0.0851 | -0.3 |
| 2 | fenvalerate | 0.1730 | 0.1973 | 0.3 |
| 3 | pirimiphos-methyl | 0.0809 | 0.0855 | 0.7 |
| 4 | Triazophos | 0.3540 | 0.3333 | 0.1 |
| **Leek homogenate** EURL-PT-FV-12, 2010 | | | | |
| 1 | azinphos-methyl | 0.048 | 0.051 | 0.3 |
| 2 | chlorothalonil | 0.216 | 0.326 | 1.9 |
| 3 | Chlorpyrifos | 0.176 | 0.200 | 0.5 |
| 4 | Ethion | 0.071 | 0.067 | -0.2 |
| 5 | fenpropathrin | 0.062 | 0.066 | 0.3 |
| 6 | kresoxim-methyl | 0.316 | 0.338 | 0.3 |
| **Cauliflower homogenate** EURL-PT-FV-11, 2009 | | | | |
| 1 | azinphos-methyl | 0.355 | 0.5057 | 1.7 |
| 2 | Boscalid | 0.414 | 0.384 | -0.3 |
| 3 | Buprofezin | 0.638 | 0.7406 | 0.6 |
| 4 | Carbofuran | 0.283 | 0.23 | -0.7 |
| 5 | deltamethrin | 0.157 | 0.2189 | 1.6 |
| 6 | diazinon | 1.250 | 1.64 | 1.2 |
| 7 | isofenphos-methyl | 0.540 | 0.5466 | 0.0 |
| 8 | lambda cyhalothrin | 0.266 | 0.3904 | 1.9 |
| 9 | metalaxyl | 0.450 | 0.44 | -0.1 |
| 10 | methidathion | 0.472 | 0.5636 | 0.8 |
| 11 | phosalone | 0.368 | 0.5046 | 1.5 |
| 12 | procymidone | 0.780 | 0.815 | 0.2 |
| 13 | triazophos | 0.538 | 0.5356 | 0.0 |
| **Tomato Purée** FAPAS, 2008 | | | | |
| 1 | parathion-methyl | 0.1569 | 0.1758 | 0.6 |
| 2 | penconazole | 0.0754 | 0.0778 | 0.1 |
| 3 | trifloxystrobin | 0.2221 | 0.2369 | 0.3 |
| 4 | vinclozolin | 0.1758 | 0.1915 | 0.4 |
| **Tomato Purée** FAPAS, 2006 | | | | |
| 1 | iprodione | 0.772 | 0.850 | 0.6 |
| 2 | metalaxyl | 0.186 | 0.220 | 0.9 |
| 3 | pirimphos-methyl | 0.262 | 0.230 | -0.6 |
| 4 | quintozene | 0.079 | 0.085 | 0.3 |
| 5 | tecnazene | 0.086 | 0.078 | -0.4 |
| **Spinach Purée** FAPAS, 2006 | | | | |
| 1 | p.p’ DDE | 0.044 | 0.048 | 0.3 |
| 2 | fenvalerate | 0.257 | 0.336 | 1.6 |
| 3 | gamma-HCH | 0.053 | 0.053 | 0.0 |
| 4 | permethrin | 0.104 | 0.116 | 0.5 |
| 5 | tolclofos-methyl | 0.151 | 0.169 | 0.6 |

**Fig. S1** Greenhouse scheme.

|  | Amistar Opti 480 SC | | | Signum 33 WG | | | Switch 62.5 WG | | | Without application |
| --- | --- | --- | --- | --- | --- | --- | --- | --- | --- | --- |
| Single dose | M_Fa_1' | M_Fa_1'' | M_Fa_1''' | M_Fb_1' | M_Fb_1'' | M_Fb_1''' | M_Fc_1' | M_Fc_1'' | M_Fc_1''' | Control |
|  | H_Fa_1' | H_Fa_1'' | H_Fa_1''' | H_Fb_1' | H_Fb_1'' | H_Fb_1''' | H_Fc_1' | H_Fc_1'' | H_Fc_1''' | Control |
| Double dose | M_Fa_2' | M_Fa_2'' | M_Fa_2''' | M_Fb_2' | M_Fb_2'' | M_Fb_2''' | M_Fc_2' | M_Fc_2'' | M_Fc_2''' | Control |
|  | H_Fa_2' | H_Fa_2'' | H_Fa_2''' | H_Fb_2' | H_Fb_2'' | H_Fb_2''' | H_Fc_2' | H_Fc_2'' | H_Fc_2''' | Control |

M - variety *Marissa*, H - variety *Harzfeuer*, 1 - single dose, 2 - double dose, ' - first replicate '' - second replicate, ''' - third repliacte, Fa - fungicide a - Amistar Opti 480 SC (azoxystrobin, chlorothalonil); Fb - fungicide b - Signum 33 WG (boscalid, pyraclostrobin); Fc - fungicide c - Switch 62.5 WG (cyprodinil, fludioxonil)

**Graphical abstract**


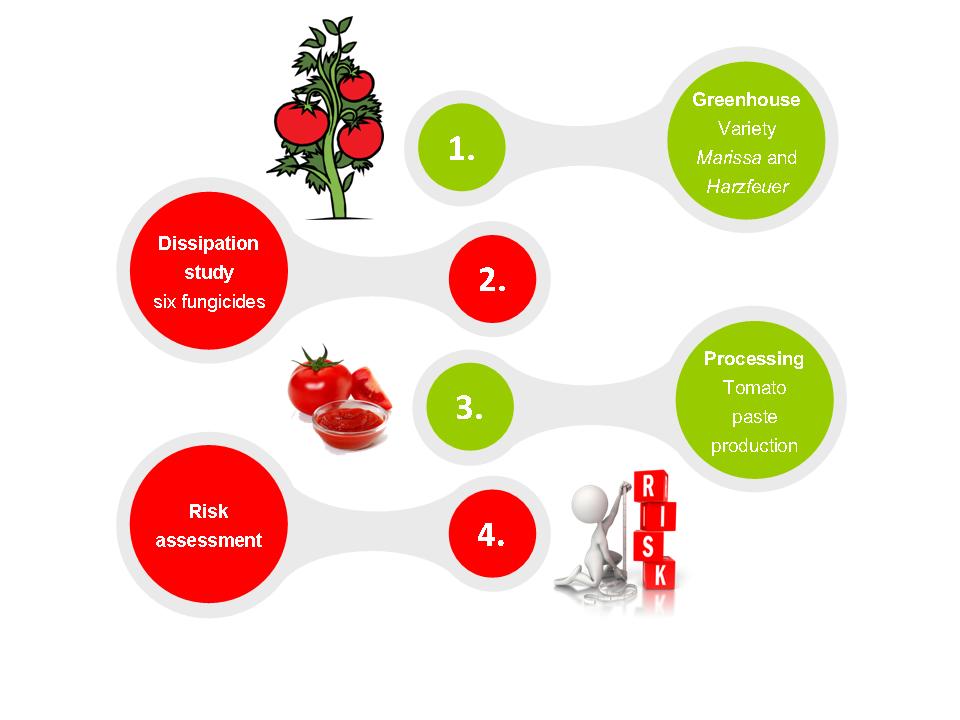

Supplement: Supplementary file 1 — Table S1. Physico-chemical properties of pesticides used in this experiment. Table S2. Health effects of active substances. Table S3. Validation parameters for tomatoes. Table S4. Analytical quality check in vegetable matrices. Figure S1. Greenhouse scheme. Graphical abstract (DOC 408 kb) [file 11356_2016_6260_MOESM1_ESM.doc]
